# Ginkgolide C Improves Cardiac Function and Ameliorates Myocardial Ischemia/Reperfusion Injury by Regulating PI3K/Akt Pathway

**DOI:** 10.33549/physiolres.935572

**Published:** 2025-12-01

**Authors:** Yanling HAO, Jinxia WU, Zheng GONG, Wenxiu ZHOU, Zhenyu WANG, Kexue LI, Tao DONG, Xia QIN, Ying WANG

**Affiliations:** 1 National Demonstration Center for Experimental Basic Medical Science Education, Xuzhou Medical University, Xuzhou, China; 2 Department of Physiology, Xuzhou Medical University, Xuzhou, China; 3 Jiangsu Provincial Key Laboratory of Anesthesiology, Xuzhou Medical University, Xuzhou, China; 4 Department of Pathophysiology, Xuzhou Medical University, Xuzhou, China

**Keywords:** Ginkgolide C, Ischemia/reperfusion injury, Cardioprotection, PI3K/Akt pathway

## Abstract

Ischemia/reperfusion (I/R) injury causes myocardial damage, and Ginkgolide C (GC), a component of *Ginkgo biloba*, shows potential for cardioprotection. However, its effects on I/R-induced cardiac dysfunction and mechanisms are unclear. This study evaluates GC preconditioning in rats, focusing on its impact on cardiac function, myocardial injury, and the PI3K/Akt pathway. GC preconditioning was assessed using an isolated rat heart model of I/R injury. Cardiac function (LVDP, LVEDP, +dp/dt_max_, -dp/dt_max_), infarct size, histopathological changes, injury, and oxidative stress markers were measured. Rat cardiomyocytes were treated with GC to assess viability, contraction, and apoptosis. Molecular docking and protein analysis explored GC’s interaction with the Akt pathway. GC preconditioning significantly improved cardiac function, with a marked enhancement in LVDP, +dp/dt_max_, and -dp/dt_max_ in the GC50 group compared to the I/R group (*P*<0.01). GC treatment also reduced myocardial infarct size (14.8 %±2.4 % vs. 25.5 %±1.9 %, *P*<0.01), decreased LDH release, and alleviated histopathological damage, including myocyte necrosis and inflammatory infiltration. Furthermore, GC significantly improved cardiomyocyte viability and contraction amplitude, particularly at 50 μM. At the molecular level, GC activated the Akt pathway, as evidenced by increased p-Akt expression. Co-treatment with the PI3K inhibitor LY294002 reversed the protective effects of GC, confirming the involvement of the Akt pathway. Additionally, GC preconditioning reduced oxidative stress, as indicated by enhanced SOD activity and decreased levels of myocardial injury markers (LDH, CK-MB), and decreased apoptosis, reflected by a lower Bax/Bcl-2 ratio. GC preconditioning reduces I/R-induced myocardial injury in rats by activating the PI3K/Akt pathway, reducing oxidative stress, inhibiting apoptosis, and improving cell viability. These results support GC’s potential for treating ischemic heart disease and suggest Akt-targeted interventions for myocardial protection.

## Introduction

Cardiovascular diseases, particularly myocardial ischemia/reperfusion (I/R) injury, remain among the leading causes of morbidity and mortality globally. While reperfusion is essential for salvaging ischemic myocardium and preventing irreversible damage, it paradoxically contributes to additional myocardial injury. This phenomenon, known as myocardial ischemia/reperfusion (I/R) injury, leads to adverse cardiovascular outcomes, including cardiac dysfunction, arrhythmias, and coronary artery spasm. These detrimental effects are primarily due to a cascade of pathological events, including oxidative stress, inflammation, calcium overload, mitochondrial dysfunction, and autophagy, which occur during the reperfusion process [1–3]. Consequently, understanding and mitigating I/R injury has become a focal point of cardiovascular research aimed at improving myocardial recovery and function.

Ginkgolide C (GC), a potent bioactive compound derived from Ginkgo biloba extract (GBE), has gained significant attention for its cardioprotective effects. GBE, a traditional herbal medicine widely used in Asia, has a long-standing history of clinical use in treating various cardiovascular diseases [4]. Among its bioactive constituents, ginkgolides, including GC, have demonstrated substantial anti-inflammatory, antioxidant, and cardioprotective properties. Clinical preparations of GBE, such as Shuxuening injection (SXNI), have been shown to have beneficial effects in treating ischemic stroke and heart diseases. GC, in particular, has been recognized for its ability to inhibit platelet aggregation [5], alleviate myocardial inflammation [6,7], promote lipolysis [8], reduce lipid accumulation [9], and even exhibit anti-tumor effects [10].

GC is a diterpene lactone characterized by a rigid hexacyclic structure comprising three fused five-membered rings and a tert-butyl group at one of the rings. Its chemical formula is C_2_0H_24_O_11_, and it exhibits unique pharmacological properties distinct from other ginkgolides [11]. Structurally, ginkgolides A, B, C, and J are similar, differing primarily in the presence or absence of hydroxyl (OH) groups at specific positions on the spirononane framework. Ginkgolide B has OH groups at positions C1 and C3, while ginkgolide C has an OH group at position C7 [12,13]. This subtle structural variation imparts distinct biological activities to GC, differentiating its effects from those of Ginkgolide B (GB).

Our previous research has demonstrated the protective role of GB in ischemic hearts, particularly in improving cardiac systolic function and reducing apoptosis [14]. However, whether GC, with its structural similarities to GB, exerts similar protective effects remains unclear. Thus, the present study aims to investigate the effects of GC preconditioning on rat cardiac function in an in vitro I/R injury model and explore its underlying mechanisms.

The phosphoinositide 3-kinase (PI3K)/Akt pathway plays a pivotal role in cellular growth, survival, and response to stress. As one of the most potent pro-survival signaling pathways, activation of the PI3K/Akt cascade facilitates cell survival by phosphorylating and activating a range of downstream targets, including key members of the Bcl-2 family (Bak, Bax, BAD), caspase-9, NF-κB, and endothelial nitric oxide synthase (eNOS) [15–17]. In the context of myocardial I/R injury, activation of the PI3K/Akt pathway has been shown to protect cardiomyocytes by inhibiting apoptosis, reducing oxidative stress, and suppressing the inflammatory response [18,19]. Recent studies have confirmed the critical role of PI3K/Akt signaling in myocardial protection against I/R injury [20,21]. However, the precise mechanisms by which GC may activate or regulate this pathway to confer cardioprotection remain unknown.

Therefore, this study investigates the hypothesis that GC preconditioning improves cardiac function and ameliorates myocardial I/R injury by modulating the PI3K/Akt signaling pathway. To assess this, we employed LY294002, a specific PI3K/Akt pathway inhibitor, to explore whether the cardioprotective effects of GC are mediated through the activation of this pro-survival signaling cascade. This work aims to provide new insights into the therapeutic potential of GC for mitigating myocardial I/R injury, with a focus on the PI3K/Akt pathway as a key regulator of cardiac function and survival.

## Materials and Methods

### Establishment of molecular docking

The core GC target associated with the active ingredient for myocardial ischemia (MI) treatment was first identified. The 2D and 3D structures of the active ingredient were retrieved from PubChem and the PDB database, respectively. Using PyMOL, water molecules and small ligands were removed. Both the protein and drug components were then converted to PDBQT format using AutoDock tools, and active binding sites were identified.

### Animals and materials

The experiments were approved by the Experimental Animal Centre of Xuzhou Medical University, and all protocols involving animals were authorized by the Animal Ethics Committee of Xuzhou Medical University. Male Sprague–Dawley (SD) rats (Clean grade, Xuzhou Medical University, China, license number: SYXK(Su) 2015-0030), aged 6–8 weeks, weighing 230–250 g, were housed in a controlled environment at room temperature (24 °C) and humidity (45–55 %) with a 12-hour light-dark cycle, and had free access to food and water.

Ginkgolide C (GC) was purchased from Sigma-Aldrich (Fluka, Germany) and dissolved in dimethyl sulfoxide (DMSO) to prepare a stock solution, with a final concentration of 0.01 % in the buffer or culture medium. Collagenase type II was obtained from Gibco (Invitrogen). Lactate dehydrogenase (LDH), superoxide dismutase (SOD), and creatine kinase-MB (CK-MB) activity assay kits were sourced from Nanjing Jiancheng Bioengineering Institute (Nanjing, China). 2,3,5-Triphe-nyl-2H-tetrazolium chloride (TTC) was purchased from Solarbio (Beijing, China). LY294002 (LY), a PI3K/Akt pathway inhibitor, was obtained from Sigma-Aldrich (St. Louis, MO, USA).

### Ischemia-reperfusion injury model and experimental groups

The I/R injury model was established in isolated rat hearts, as described previously [14]. Male Sprague–Dawley rats were anesthetized and pretreated with heparin, and hearts were excised and perfused in a Langendorff apparatus with Krebs-Henseleit buffer at 80 mmHg. After a 20-minute stabilization, hearts were randomly assigned to five groups (6 hearts per group): Control (normal perfusion), I/R (30 min ischemia followed by 60 min reperfusion), GC25, GC50, and GC100 (GC preconditioning at 25, 50, and 100 μM, respectively, for 5 min before ischemia). Additionally, a GC50+LY group was treated with 50 μM GC and 10 μM LY294002 (PI3K/Akt inhibitor). Coronary effluent was collected at the 5th min of reperfusion for LDH activity analysis, and hearts were stored at −20 °C for further infarct size and histological assessment. All experiments were conducted in six times to ensure reproducibility and statistical reliability.

### Infarct size assessment

Myocardial infarct size was evaluated using the triphenyltetrazolium chloride (TTC) staining method. After reperfusion, hearts were frozen and sliced into 2-mm transverse sections. These slices were incubated in 2 % TTC at 37 °C for 15 minutes, fixed, and photographed. Infarcted areas appeared pale, while viable tissue was stained red. The infarct area of each slice was measured using computerized planimetry (Image-Pro Plus software), and the infarct volume was calculated by multiplying the infarct area by the slice weight. The total infarct size was then expressed as a percentage of the total left ventricular volume.

### Hematoxylin and Eosin (H&E) Staining

After reperfusion, hearts were collected and fixed in 4 % paraformaldehyde, followed by paraffin embedding. Cross-sectional slices of the heart were then deparaffinized, rehydrated, and stained with hematoxylin and eosin (HE). Images were captured using a microscope (IX 71, OLYMPUS, Japan).

### Isolation of rat ventricular myocytes and simulated I/R injury

Ventricular myocytes were isolated and I/R injury models were simulated as previously described. Isolated rat hearts(n=6) were retroperfused with a Ca^2+^-free buffer for 5 minutes, followed by cyclic perfusion with the same buffer supplemented with 50 μM CaCl_2_, 0.04 % collagenase, and 0.1 % bovine serum albumin. After approximately 25 minutes, the hearts became soft, and the left ventricle was excised, minced in Krebs–bicarbonate (KB) solution, and single myocytes were filtered through a 200-mesh nylon filter. The viable myocytes were harvested by resuspending them in KB solution three times. Myocytes were cultured in Dulbecco’s minimal essential medium (DMEM) at a density of 2×10^4^ cells per well in 12-well culture dishes.

Cardiomyocytes were incubated in a cell culture chamber at 37 °C with 5 % CO_2_ and 95 % air. The cells were divided into the following groups: Control, I/R, and GC subgroups. In the Control group, cardiomyocytes were cultured normally for 7 hours. In the I/R group, after 2 hours of normal culture, the cells were subjected to ischemic conditions by replacing the culture medium with ischemic buffer (containing 118 mM NaCl, 24 mM NaHCO_3_, 1.0 mM NaH_2_PO_4_, 2.5 mM CaCl_2_, 1.2 mM MgCl_2_, 20 mM sodium lactate, 16 mM KCl, and 10 mM 2-deoxyglucose, pH 6.2) and transferred to a tri-gas incubator (1 % O_2_, 5 % CO_2_, 94 % N_2_) at 37 °C for 3 hours. Reperfusion was initiated by replacing the ischemic buffer with normal culture medium and returning the cells to the normal incubator for 2 hours, simulating I/R injury.

In the GC subgroups, cardiomyocytes were cultured with varying concentrations of GC (1, 10, 50, 100, and 200 μM) for 2 hours, followed by simulated I/R treatment. The optimal GC concentration of 50 μM was identified based on subsequent measurements of cardiomyocyte shortening amplitude. In the GC+LY and LY groups, 50 μM LY294002 (a PI3K/Akt inhibitor) was added to the culture medium, with or without 50 μM GC, followed by simulated I/R treatment. After reperfusion, the culture medium was collected to assess LDH and CK-MB activity, and cardiomyocytes were collected for SOD activity measurements.

### Measurement of cardiomyocyte contraction amplitude

After normal culture or I/R protocol, a few drops of the medium containing myocytes were placed in an open chamber on the stage of an inverted microscope (Olympus, Tokyo, Japan). Five minutes later, the myocytes spontaneously settled at the bottom of the chamber, which was then filled with Krebs-Henseleit buffer (KHB) containing 2 mM Ca^2+^ and 100 nM isoprenaline. Electrical field stimulation was applied at 0.5 Hz. Myocardial contraction was recorded using a video edge detector system (Panasonic, Osaka, Japan) and analyzed using Optical Measure software (China’s National Defense University of Science and Technology, Changsha, China).

### Determination of cardiomyocyte survival rate

Viable cardiomyocytes appear rod-shaped, while nonviable cells are round. The cardiomyocytes were counted under an inverted microscope, and five random micrographs were taken for each sample. All myocytes in these fields were assessed under a single preparation condition. The survival rate was calculated as the percentage of rod-shaped cells relative to the total number of cells.

### Measurement of cardiac injury and oxidative stress markers

Cardiac injury markers, such as LDH and CK-MB, were measured in the coronary effluent or culture medium to assess myocardial damage, using the AU5800 automatic biochemical analyzer (Beckman Coulter). Additionally, oxidative stress was evaluated by measuring SOD activity in the homogenized cardiomyocytes from each group. The assays were performed according to the manufacturer’s instructions.

### Western Blot

Following normal culture or I/R protocol, cardiomyocytes were collected and centrifuged at 500 g for 1 min, then resuspended in phosphate-buffered saline. Cells were lysed in a solution containing 15 % sodium dodecyl sulfate, 100 mM Tris–HCl (pH 6.8), 40 mM phenylmethylsulfonyl fluoride, and 10 mM EDTA, with vortexing every 5 min for 5 cycles. After centrifugation at 13,000 rpm for 15 min, the supernatant was collected for protein quantification. Proteins were separated by 10 % SDS-polyacrylamide gel electrophoresis and transferred to a nitrocellulose membrane. The membrane was blocked with 4 % non-fat milk and incubated overnight at 4 °C with primary antibodies (1:1000 dilution) against Bcl-2, Bax, and p-Akt (Cell Signaling Technology, Beverly, MA, USA). After three 5-min washes in Tris-buffered saline/Tween 20, the membrane was incubated with the corresponding secondary antibodies (1:5000, Bioworld, China) for 2 hours at room temperature. Following five 3-min washes, protein bands were developed using nitro blue tetrazolium and 5-bromo-4-chloro-3-indolyl-phosphate. The membrane was scanned, and the relative intensity of the bands was analyzed using Adobe Photoshop software (San Jose, CA, USA). All Western blot experiments were conducted in triplicate.

### Statistical analyses

Data from each experimental series are presented as mean ± SEM. Statistical analysis was performed using GraphPad Prism software (Version 5.0). One-way analysis of variance (ANOVA) followed by Bonferroni correction for multiple comparisons was used to assess statistical significance (*P* < 0.05) for each variable.

## Results

### GC preconditioning ameliorated cardiac function in rats following I/R injury

**Fig. 1:**
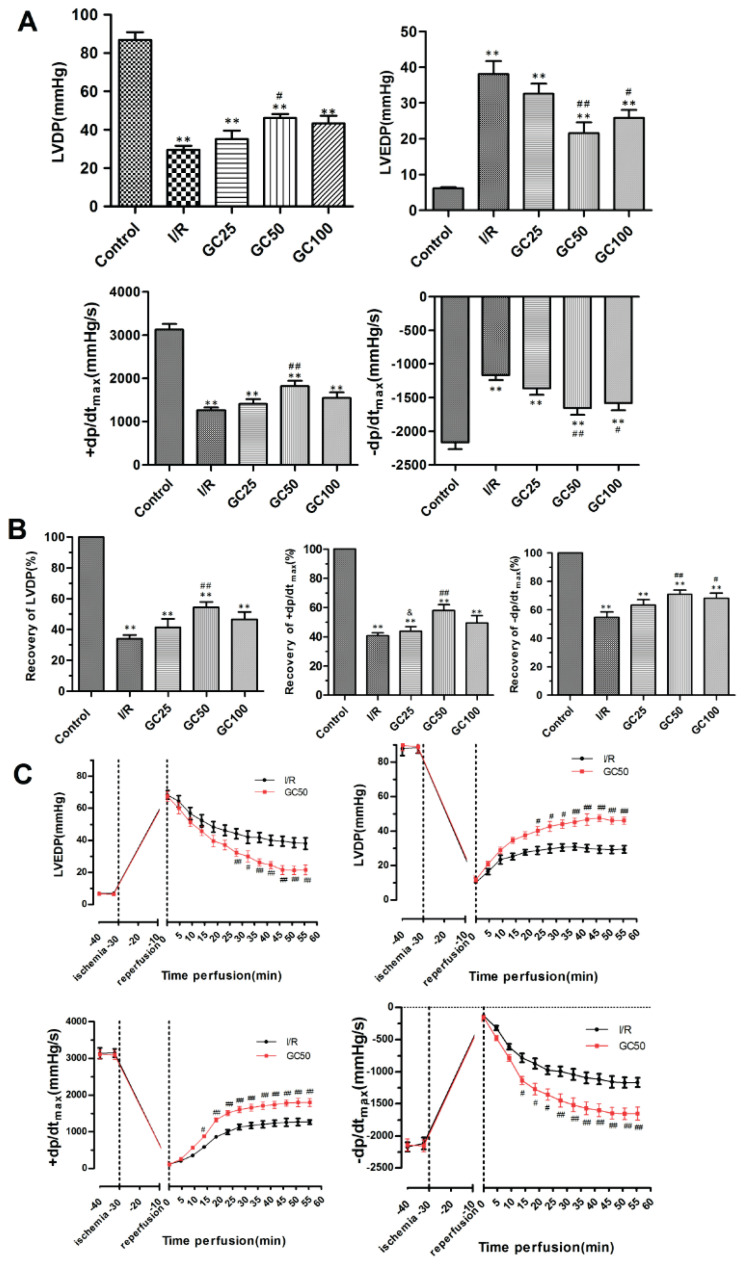
Effects of GC preconditioning on I/R induced cardiac dysfunction in rats. (**A**) Hemodynamic parameters recorded after 60 min perfusion. (**B**) The recovery rates of hemodynamic parameters at 60 min perfusion. (**C**) Continuous hemodynamic parameters in I/R group and GC50 group. Control, 90 min perfusion without ischemia. I/R, 30 min ischemia followed by 60 min reperfusion. Different concentration GC subgroups (GC25, GC50 and GC100) 25, 50 and 100 μM ginkgolide C was perfused for 5 min before ischemia, respectively. n=6 rats per group. Data are presented as mean ± SEM. **P<0.01 vs control. ^#^*P*<0.05, ^##^*P*<0.01vs I/R.

The cardiac function parameters, including Left Ventricular Developed Pressure (LVDP), Left Ventricular End-Diastolic Pressure (LVEDP), the rate of pressure rise (+dp/dt_max_), and the rate of pressure decline (-dp/dt_max_), measured after 60 min of reperfusion, are shown in Fig. 1A. Compared to the control group, all parameters in the I/R group and GC treatment groups showed statistically significant differences. Notably, the GC50 group demonstrated significant improvements in all parameters compared to the I/R group: LVDP (46.05±2.07 mmHg vs. 29.44±2.12 mmHg, *P*<0.05), LVEDP (21.57±3.03 mmHg vs. 38.07±3.57 mmHg, *P*<0.01), +dp/dt_max_ (1823.3±118.1 mmHg/s vs. 1264±59.4 mmHg/s, *P*<0.01), and −dp/dt_max_ (−1653±102.1 mmHg/s vs. −1165±72.6 mmHg/s, *P*<0.01). Additionally, the GC100 group showed a significant reduction in LVEDP and −dp/dt_max_ (*P*<0.05 vs. I/R). However, there were no significant differences in any of the parameters in the GC25 group.

In terms of recovery (Fig. 1B), the GC50 group exhibited markedly better recovery rates than the I/R group: LVDP (54.32 %±3.62 %), +dp/dt_max_ (58.08 % ±3.98 %), and −dp/dt_max_ (76.98 %±4.75 %) were significantly higher (*P*<0.01). These findings indicate that 50 μM GC significantly improved cardiac systolic function.

Throughout the entire perfusion period (Fig. 1C), cardiac function parameters were significantly lower post-ischemia than pre-ischemia but gradually improved during reperfusion. After 30 min of reperfusion, the GC50 group showed significant improvements in all cardiac function parameters compared to the I/R group at the same time point. Overall, these results demonstrate that 50 μM GC can alleviate the decline in cardiac function and improve overall cardiac performance following ischemia/reperfusion injury.

### GC preconditioning reduced infarct size, myocardial injury and histopathological changes in Rats following I/R injury

**Fig. 2:**
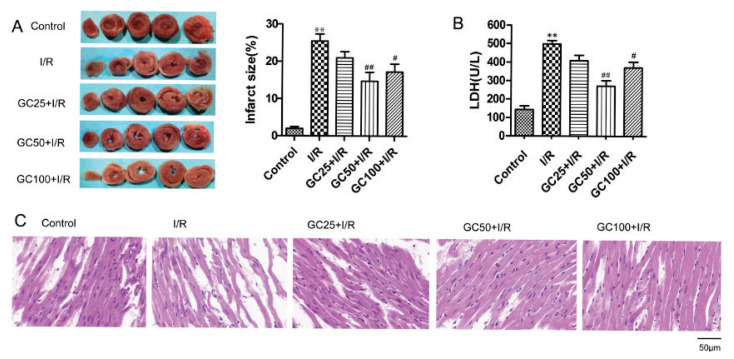
Effects of GC preconditioning on infarct size, myocardial injury and histopathological changes following I/R in rats (n=6). (**A**) Infarct size in each group after 60 min reperfusion. (**B**) Effect of GC on the release of LDH in coronary effluent after ischemia. (**C**) H&E staining of myocardial histology (scale bar = 50μm). Data are presented as mean ± SEM. **P<0.01 vs control. ^#^*P*<0.05, ^##^*P*<0.01vs I/R.

In the I/R group, irreversible I/R injury resulted in an infarct size of 25.5 %±1.9 %, which was significantly higher compared to the control group (*P*<0.01). In contrast, 50 μM GC treatment significantly reduced the infarct size caused by I/R injury to 14.8 %±2.4 % (*P*<0.01), indicating that GC can effectively reduce the myocardial infarction size following I/R injury (Fig. 2A).

LDH levels in the coronary effluent of the I/R group were significantly higher than those in the control group (Fig. 2B). Treatment with GC, particularly the 50 μM dose (GC50), resulted in a significant reduction in LDH content compared to the I/R group (*P*<0.01), suggesting that GC helps decrease myocardial cell damage induced by I/R injury.

In the control group, H&E staining showed that myocytes were regularly arranged, with clear nuclei and no inflammatory cells in the myocardial interstitium (Fig. 2C). In contrast, the I/R group displayed irregularly arranged myocytes, uneven staining, areas of necrosis, and fragmented myocardial cells, along with an infiltration of inflammatory cells in the interstitium. GC preconditioning significantly reduced these histological changes, preserving the regular arrangement of myocytes and reducing inflammatory cell infiltration.

### GC preconditioning increased the shortening amplitude, viability and decreased apoptosis of cardiomyocytes

**Fig. 3:**
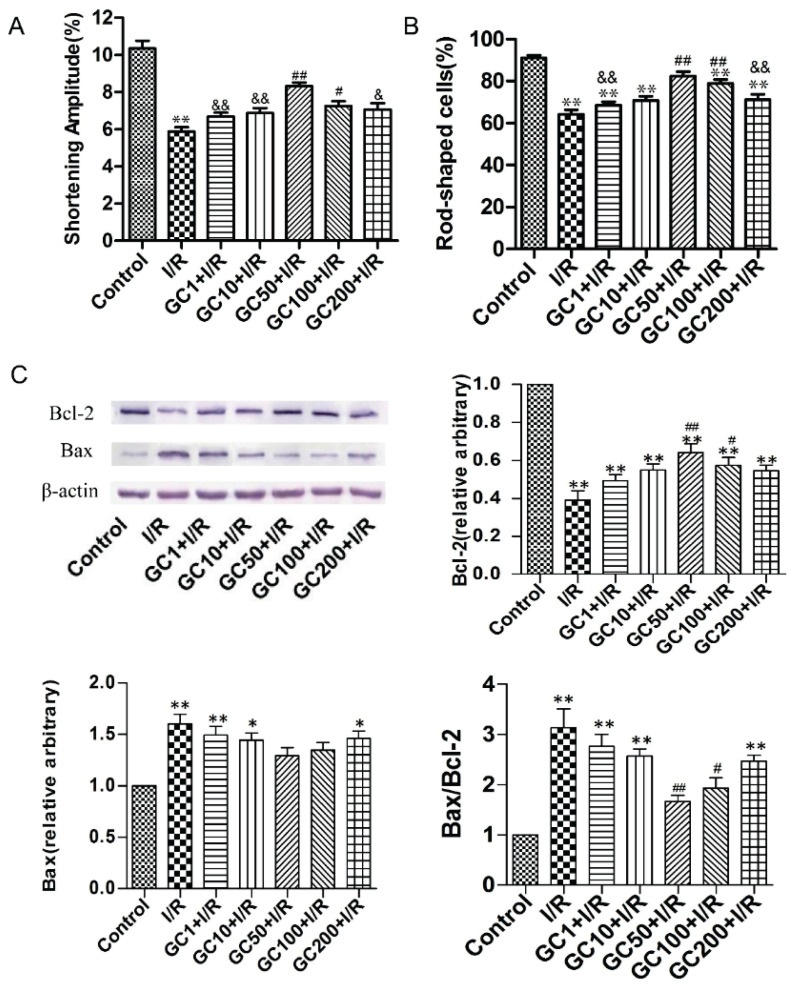
Effects of GC on myocytes shortening amplitude, viability and apoptosis after I/R culture. (**A**) Effect of GC on shortening amplitude of myocytes (n=6). (**B**) Effect of GC on the survival of myocytes (n=6). (**C**) Effects of GC on myocytes apoptosis (n=3). Data are presented as mean ± SEM. *P<0.05, **P<0.01 vs Control. ^#^*P*<0.05, ^##^*P*<0.01 vs I/R. ^&^*P*<0.05 and ^&&^*P*<0.01 vs GC50. Control, without simulative I/R treatment. I/R, myocytes were subjected to 3 h ischemia followed by 2 h reperfusion. GC subgroups (GC1, GC10, GC50, GC100 and GC200), myocytes were cultured respectively with different concentrations of ginkgolide C at 1, 10, 50, 100 and 200 μM before ischemia for 2 h.

In the I/R group, the shortening amplitude of cardiomyocytes was significantly reduced compared to the control group. However, treatment with 50 μM GC (GC50) and 100 μM GC (GC100) resulted in a noticeable increase in the shortening amplitude compared to the I/R group (GC50: 8.3 %±0.2 %, *P*<0.01; GC100: 7.3 %±0.2 %, *P*<0.05 vs. I/R: 5.8 %±0.2 %). Notably, 50 μM GC effectively inhibited the decline in cardiomyocyte contraction induced by I/R, with GC50 being the most effective concentration (Fig. 3A). These findings suggest that GC preconditioning can prevent the attenuation of cardiomyocyte contraction caused by I/R injury, with 50 μM GC being the optimal dose.

Additionally, the percentage of rod-shaped cells was significantly reduced in the I/R group compared to the control group. However, in the GC50 and GC100 groups, the percentage of rod-shaped cells increased markedly compared to the I/R group, with the GC50 group showing a more pronounced improvement (GC50: 80.5 %±2.4 % vs. I/R: 63.0 %±2.5 %, *P*<0.01, Fig. 3B). This indicates that GC preconditioning, especially at the 50 μM concentration, significantly enhances cardio-myocyte viability following I/R injury, highlighting its protective effect.

Furthermore, apoptosis analysis *via* the Bax/Bcl-2 ratio showed a marked decrease in apoptosis in the GC50 and GC100 groups compared to the I/R group. This indicates that GC preconditioning reduces apoptosis, with 50 μM GC exhibiting the strongest protective effect. Overall, GC preconditioning not only improves cardiomyocyte contraction but also enhances cell viability and reduces apoptosis following I/R injury (Fig. 3C).

### GC interacted with Akt to improve myocyte shortening amplitude and viability following I/R injury

**Fig. 4:**
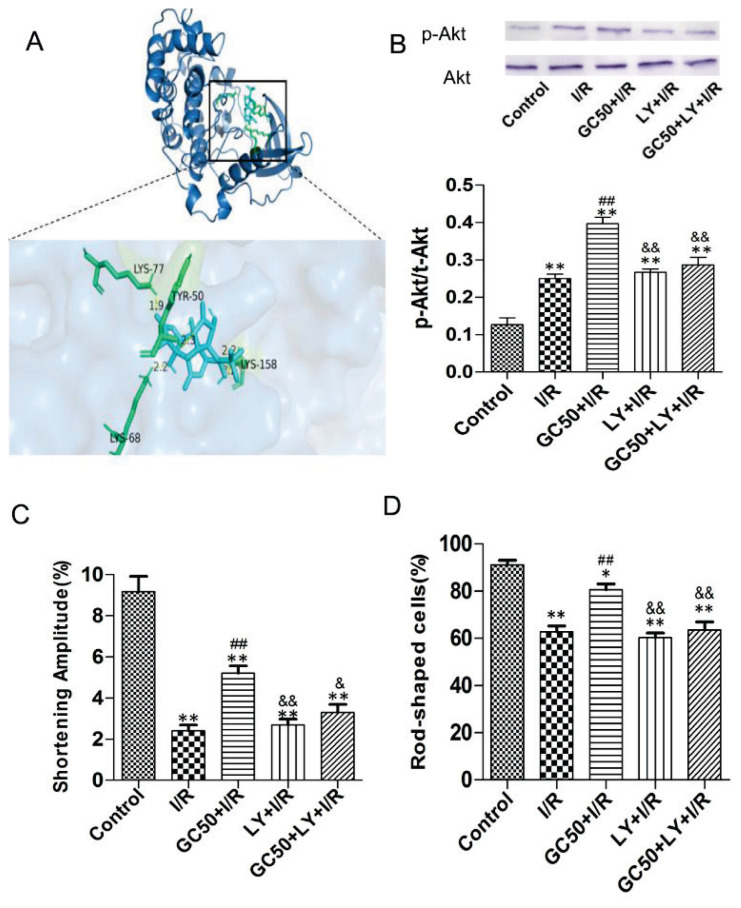
The interaction between GC and Akt and their effects on myocytes shortening amplitude and viability following I/R. (**A**) Molecular docking simulation results of GC and Akt. (**B**) Akt and p-Akt protein expression (n=3). (**C**) Effect of GC and LY on shortening amplitude of myocytes (n=6). (**D**) Effect of GC and LY on myocytes survival (n=6). Data are presented as mean ± SEM. **P<0.01 vs Control. ^##^*P*<0.01 vs I/R. ^&^*P*<0.05 and ^&&^*P*<0.01 vs GC50+I/R. GC50+LY+I/R, myocytes were cultured with both 50 μM ginkgolide C and LY294002 before ischemia for 2 h.

Molecular docking simulations revealed a strong binding affinity between GC and Akt (Fig. 4A), suggesting that GC may modulate Akt signaling to exert its protective effects on cardiomyocytes. Protein expression analysis showed that GC50 treatment significantly increased the levels of phosphorylated Akt (p-Akt) compared to the I/R group (Fig. 4B), indicating activation of the Akt pathway by GC. Conversely, treatment with the PI3K inhibitor LY294002 (LY) reduced p-Akt levels in both the I/R and GC50+I/R groups, confirming that LY294002 effectively inhibits the PI3K/Akt pathway.

The effect of GC on myocyte shortening amplitude was evaluated in the presence or absence of the PI3K inhibitor, LY294002. The I/R group exhibited a significant reduction in shortening amplitude compared to the control group. Treatment with GC50 (GC50+I/R) significantly improved the shortening amplitude compared to the I/R group. However, co-treatment with LY294002 (GC50+LY+I/R) inhibited this improvement, suggesting that the protective effect of GC on myocyte contraction is at least partially mediated through Akt activation (Fig. 4C).

Cardiomyocyte viability was assessed by quantifying the percentage of rod-shaped cells, a morphology indicative of viable cardiomyocytes. The I/R group showed a marked reduction in the percentage of rod-shaped cells compared to the control group. Treatment with GC50 (GC50+I/R) significantly increased the percentage of rod-shaped cells compared to the I/R group. Co-treatment with LY294002 (GC50+LY+I/R) attenuated the protective effect of GC on cell viability. These findings further support the conclusion that GC enhances myocyte viability through Akt activation (Fig. 4D).

Collectively, these results demonstrate that GC interacts with the Akt pathway to improve both myocyte shortening amplitude and viability following I/R injury, and that this protective effect is diminished when Akt signaling is inhibited.

### GC preconditioning significantly mitigated myocardial injury, oxidative stress, and apoptosis via the Akt pathway following I/R in rats

**Fig. 5:**
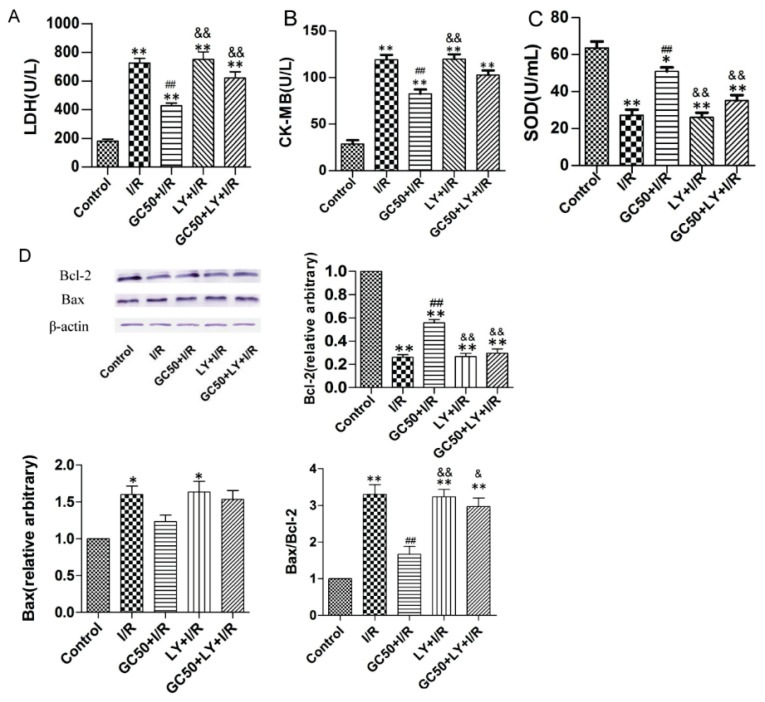
Effects of GC preconditioning on myocardial injury, oxidative stress, and apoptosis via Akt pathway following I/R in rats. (**A–B**) Assessment of myocardial injury markers (LDH and CK-MB) (n=6). (**C**) Assessment of myocardial oxidative stress marker, SOD (n=6). (**D**) Bcl-2 and Bax protein expression in each group myocytes after simulative I/R injury (n=3). Data are presented as mean ± SEM. **P<0.01 vs control. ^#^*P*<0.05, ^##^*P*<0.01 vs I/R, ^&^*P*<0.05 and ^&&^*P*<0.01 vs GC50+I/R.

As shown in Fig. 5A–C, the I/R group exhibited elevated levels of myocardial injury markers, including lactate dehydrogenase (LDH) and creatine kinase-MB (CK-MB), and decreased activity of superoxide dismutase (SOD), a key antioxidant enzyme. Treatment with 50 μM GC (GC50) notably reduced LDH and CK-MB levels and enhanced SOD activity compared to the I/R group (*P*<0.01). However, the increase in SOD activity was partially inhibited by the PI3K inhibitor LY294002 (LY) in the GC50+LY group, indicating that the protective effects of GC on oxidative stress are at least partially mediated through the Akt pathway.

Regarding apoptosis, as depicted in Fig. 5D, the I/R group showed a significant increase in the Bax/Bcl-2 ratio, a marker of apoptosis. GC preconditioning (GC50 and GC100 groups) significantly decreased the Bax/Bcl-2 ratio compared to the I/R group, suggesting a reduction in apoptosis. This effect was attenuated in the GC50+LY group, where the Bax/Bcl-2 ratio was significantly higher than in the GC50 group, indicating that the anti-apoptotic effects of GC are partially mediated through the Akt pathway.

Collectively, these findings demonstrate that GC preconditioning attenuates myocardial injury, oxidative stress, and apoptosis following I/R injury in rats, with these protective effects being partially mediated through the Akt signaling pathway.

## Discussion

In this study, we have demonstrated that GC preconditioning significantly improves cardiac function and ameliorates myocardial I/R injury in rats. Our findings indicate that GC, particularly at a concentration of 50 μM, enhances cardiac function by improving key parameters such as LVDP, +dp/dt_max_, and −dp/dt_max_. These results are consistent with previous studies highlighting the cardioprotective effects of ginkgolides. For example, Ginkgolide B (GB), a structurally similar compound, has been shown to improve myocardial function and reduce infarct size in animal models of I/R injury [22]. Our study further expands on this by demonstrating the efficacy of GC in reducing infarct size and myocardial injury markers, including LDH and CK-MB, which are widely used as indicators of myocardial damage [23]. The protective effects of GC were also evident in the preservation of myocardial architecture, with histological analysis showing a reduction in myocardial cell necrosis and inflammatory cell infiltration, further emphasizing GC’s potential as a therapeutic agent for ischemic heart disease.

The mechanism underlying GC’s cardioprotective effects appears to involve the activation of the PI3K/Akt signaling pathway, a well-established pro-survival pathway critical in cellular responses to stress. Our molecular docking studies and protein expression analysis revealed that GC interacts with Akt, leading to its phosphorylation and activation, thereby triggering downstream anti-apoptotic and cytoprotective effects. Akt, regulated by PI3K, plays a key role in various cellular processes in the heart [24]. Notably, co-treatment with the PI3K inhibitor LY294002 attenuated the beneficial effects of GC on myocyte shortening amplitude and cell viability providing strong evidence that the protective effects of GC are mediated, at least in part, through Akt signaling. These results corroborate findings from other studies in which PI3K/Akt activation was shown to play a pivotal role in reducing myocardial damage during I/R injury [25,26].

Numerous studies have demonstrated that activating the Akt pathway offers protective effects against I/R injury across multiple organs, including the heart, kidney, brain, and liver, through mechanisms such as inhibition of apoptosis, reduction of oxidative stress, and suppression of inflammation [25,27–30]. Specifically, a study has shown that Akt activation can enhance the activity of antioxidant enzymes, thereby mitigating oxidative damage [31]. Our study aligns with these findings, showing that GC preconditioning *via* Akt signaling reduces myocardial injury, oxidative stress, and apoptosis during I/R. This is demonstrated by the decreased levels of LDH and CK-MB, increased SOD activity, and a lower Bax/Bcl-2 ratio, all of which suggest that the protective effects of GC are, in part, mediated through the Akt pathway.

This finding from our study underscores the importance of Akt as a central hub in cellular defense mechanisms, not only in protecting against oxidative damage but also in maintaining the delicate balance between pro-apoptotic and anti-apoptotic signals. The reduction in the Bax/Bcl-2 ratio observed with GC treatment highlights its potential to shift the apoptotic threshold, favoring cell survival even under conditions of ischemia/reperfusion injury. Moreover, given that oxidative stress and apoptosis are tightly intertwined in the pathophysiology of I/R injury [32], this dual modulation by GC provides a promising therapeutic strategy to preserve myocardial function. The evidence from our study further advocates for exploring Akt-based interventions as potential avenues to mitigate I/R-induced cardiac injury, particularly in the context of preconditioning strategies like GC.

In addition to Akt, mTOR, a key downstream effector in the PI3K/Akt pathway, plays a pivotal role in I/R injury by regulating protein synthesis, autophagy, and cell survival. It functions through two complexes, mTORC1 and mTORC2, which are essential for adaptive cardiac responses and cardiomyocyte survival under stress. While mTORC1 activation supports myocardial protection during I/R, its partial inhibition has also been shown to reduce pathological remodeling, highlighting its dual role in cardiovascular health [33]. These findings emphasize the relevance of mTOR signaling in the cardioprotective mechanisms that may be associated with GC preconditioning.

GC preconditioning likely protects the heart through the activation of both Akt and mTOR signaling pathways, which are crucial for maintaining mitochondrial function during I/R injury. By supporting oxidative phosphorylation and ATP production, these pathways help preserve cellular energy stores and reduce oxidative stress, contributing to the observed reduction in apoptosis and myocardial injury[34]. A study has shown that GC reduced oleic acid-induced lipid accumulation in HepG2 cells, suggesting that GC may also influence lipid metabolism [9], which could further enhance its cardioprotective effects. This highlights the broader role of GC in modulating cellular processes beyond mitochondrial function, including the regulation of lipid homeostasis. Together, these mechanisms emphasize the importance of mitochondrial bioenergetics and metabolic regulation in GC’s cardioprotective effects, suggesting that GC may enhance cellular survival by stabilizing mitochondrial function and supporting metabolic balance. This mechanism further supports the therapeutic potential of GC in ischemic heart disease.

## Conclusion

**Fig. 6:**
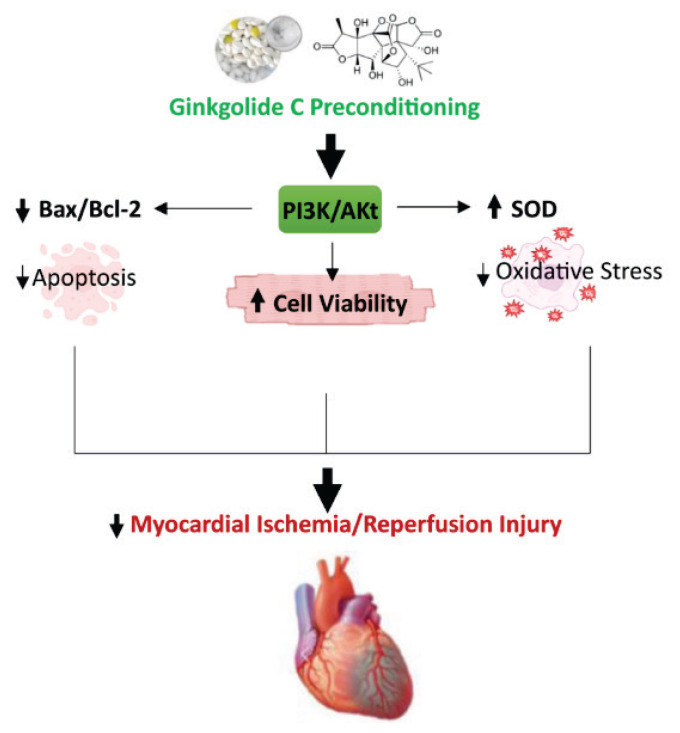
Schematic illustration of the Protective Mechanism of Ginkgolide C Preconditioning Against Myocardial Ischemia/Reperfusion (I/R) Injury. Ginkgolide C preconditioning activates the PI3K/Akt signaling pathway, leading to enhanced cell viability through multiple mechanisms. It downregulates the pro-apoptotic Bax/Bcl-2 ratio, thereby reducing apoptosis, and upregulates superoxide dismutase (SOD), decreasing oxidative stress. These combined effects attenuate myocardial ischemia/reperfusion injury, ultimately contributing to improved cardiac outcome.

In conclusion, our study demonstrates that GC preconditioning exerts a significant protective effect against myocardial I/R injury in rats, improving cardiac function, reducing infarct size, and alleviating markers of myocardial injury (Fig. 6). The cardioprotective effects of GC are primarily mediated through the activation of the PI3K/Akt signaling pathway, which plays a pivotal role in reducing oxidative stress, inhibiting apoptosis, and maintaining cellular viability during I/R injury. Our findings suggest that GC holds promise as a therapeutic agent for ischemic heart disease, offering potential benefits in preserving myocardial function through its multifaceted modulation of cellular stress responses. These results also highlight the central role of Akt signaling in protecting against myocardial damage, providing a strong rationale for further exploration of Akt-based interventions in future therapeutic strategies.

## Limitation

While our study provides compelling evidence for the cardioprotective effects of GC, several limitations should be considered. First, the experiments were conducted using a rat isolated organ model, and while this is a well-established model for I/R injury, further studies in larger animal models or human clinical trials are necessary to confirm the translational relevance of our findings. Second, although we have identified the involvement of the PI3K/Akt pathway, additional mechanistic studies are needed to explore other potential pathways that may contribute to GC’s protective effects. For instance, interactions with other pro-survival signaling networks, such as NF-κB or mTOR, were not evaluated and could provide further insight into the multifactorial nature of GC’s cardioprotective actions. In particular, future studies should investigate whether GC modulates the mTOR pathway, given its central role in cardiac physiology and stress response. Lastly, while GC shows promise as a preconditioning agent, its long-term safety and efficacy, particularly in chronic ischemic conditions, remain to be thoroughly investigated. Future studies should address these gaps to fully assess the therapeutic potential of GC in ischemic heart disease.
